# Triploidy in Chinese parthenogenetic *Helophorus orientalis* Motschulsky, 1860, further data on parthenogenetic *H. brevipalpis* Bedel, 1881 and a brief discussion of parthenogenesis in Hydrophiloidea (Coleoptera)

**DOI:** 10.3897/CompCytogen.v14i1.47656

**Published:** 2020-01-13

**Authors:** Robert B. Angus, Fenglong Jia

**Affiliations:** 1 Institute of Entomology, School of Life Sciences, Sun Yat-sen University, Guangzhou, 510275, Guangdong, China Sun Yat-sen University Guangdong China; 2 Department of Life Sciences (Insects), The Natural History Museum, Cromwell Road, London SW7 5BD, UK The Natural History Museum London United Kingdom

**Keywords:** China, Coleoptera, *Helophorus orientalis*, *H. brevipalpis*, Hydrophiloidea, karyotypes, parthenogenesis, triploidy

## Abstract

The chromosomes of triploid parthenogenetic *Helophorus orientalis* Motschulsky, 1860 are described from material from two localities in Heilongjiang, China. 3n = 33. All the chromosomes have clear centromeric C-bands, and in the longest chromosome one replicate appears to be consistently longer than the other two. The chromosomes of additional triploid parthenogenetic *H. brevipalpis* Bedel, 1881, from Spain and Italy, are described. In one Italian population one of the autosomes is represented by only two replicates and another appears more evenly metacentric than in material from Spain and the other Italian locality. Parthenogenetic and bisexual specimens of *H. orientalis* are illustrated, along with Pleistocene fossil material. Parthenogenetic *H. brevipalpis* is also illustrated. Parthenogenesis in Hydrophiloidea is discussed. It appears to be rare and, in all cases has been detected by chromosomal analysis of populations in which males are unexpectedly scarce. Parthenogenesis is suspected in *Helophorus aquila* Angus et al., 2014, from northern Qinghai (China), which should be verified in further studies.

## Introduction

The family Helophoridae is one of the basal clades of the superfamily Hydrophiloidea (Fikáček et al. 2012). The family comprises about 189 species classified in nine subgenera of which *Rhopalohelophorus* Kuwert, 1886, with about 146 species, is the largest and includes both *Helophorus brevipalpis* and *H. orientalis* (Angus 2015, Smetana 1985).The basic diploid chromosome number for *Rhopalohelophorus*, known in 31 species, is 2n = 22 (Angus 1989, 1996, 1998, 2019, Angus et al. 2005, Angus and Aouad 2009), a number shared with the family Hydrochidae, another of the basal Hydrophiloid lineages (Shaarawi and Angus 1992). The other diploid chromosome number occurring in Helophoridae is 2n = 18, found in the subgenera *Helophorus* s. str. Fabricius, 1775, *Gephelophorus* Sharp, 1915 and *Eutrichelophorus* Sharp, 1915 and occurring in many of the aquatic Hydrophilidae (Angus, 1989).

Parthenogenesis appears to be rare in the Hydrophiloidea and to date has been recorded only in *H. orientalis* Motschulsky, 1860 (Angus 1970) and *H. brevipalpis* Bedel, 1881 in the Helophoridae (Angus 1992), and *Anacaena lutescens* Stephens, 1829 in the Hydrophilidae (van Berge Henegouwen 1986, Shaarawi and Angus 1991). Angus (1992) has shown that in *H. brevipalpis* Bedel, 1881 both diploid and triploid females may coexist in one population.

The aim of the present study was to study the karyotypes of two *Helophorus* species originating from China (*H. orientalis*) and Mediterranean region (*H. brevipalpis*) and to determine the mode of reproduction of the species, bisexual or parthenogenetic, in these unstudied populations.

## Material and methods

The material used for chromosome analysis is listed in Table 1. The number of specimens refers to the number from which successful preparations were obtained. The material was collected with a water net in small pools and ditches. The *H. orientalis* was collected by Angus and Jia, the *H. brevipalpis* by Angus.

**Table 1.** The species, location of populations and the number of specimens studied.

Species	Locality	No. examined
*Helophorus orientalis* Motschulsky, 1860	China, Heilongjiang: Mishan, Dading Shan Forestry Study Centre. 45.3635N, 131.9175E	2♀♀
	China, Heilongjiang: Qitaihe, Shillongshan National Forest. 45.6409N, 131.264E	3♀♀
*Helophorus brevipalpis* Bedel, 1881	Spain, Leon: Algadefe. 42.215N, 5.590W	2♂♂, 10♀♀ (Angus 1992)
	Italy, Parma: Ponte Scipione. 44.8315N, 9.956E	1♀
	Italy, Reggio Emilia: Near Sologno. 44.375N, 10.402E	5♀♀

Following the protocol described by Angus (2006), chromosome preparations were obtained from mid-gut of adult beetles. Beetles were injected with 0.1% colchicine solution in insect saline (0.75% NaCl in distilled water buffered to pH 6.8 with Sörensen’s phosphate buffer) and left for 12.5 min. They were then transferred to a 0.48% (^1^/_2_-isotonic) solution of KCl at pH 6.8 in individual solid watch glasses, their abdomens detached, and the midguts removed and left in the solution. The rest of the beetle was removed, killed by immersion in boiling water, and mounted on a card as a voucher. After 12.5 min, the guts were transferred to fixative (3 parts of absolute ethanol and 1part of glacial acetic acid), again in solid watch glasses. The fixative was changed twice, and the guts were then left to stay in fixative for 1 hour, with the watch glasses covered to prevent water being absorbed from the air. For chromosome preparations small pieces of tissue were taken with fine forceps and placed on clean dry slides, cells were disaggregated in a small drop of 45% acetic acid, with the tissue torn apart with fine pins as necessary. Next, a drop of fixative was pipetted on to the cell suspension. This causes the drop to spread over the slide as a thin film. The spreading film can be guided by tilting the slide. Sides were dried horizontally. After at least 1 hour, they were stained with 0.5% Giemsa solution at pH 6.8.

Chromosomes were photographed under oil-immersion (X100 objective) on to high-contrast microfilm. Photographs were printed at X 3000, then scanned into a computer and further processed using Adobe Photoshop.

For C-banding the immersion oil used for photographing the preparations was removed by washing in xylene (2 changes) and absolute ethanol. The slides were then dried vertically. C-banding was done by immersing the slides in saturated barium hydroxide at room temperature for 4 minutes, followed by 1 hour in 2X SSC (Salt-Sodium Citrate: 0.3 M NaCl + 0.03 M trisodium citrate) at 60⁰C. The C-banding protocol could be repeated if initial results were not satisfactory.

## Results

### *H. orientalis* Motschulsky, 1860

**Figure 1.**
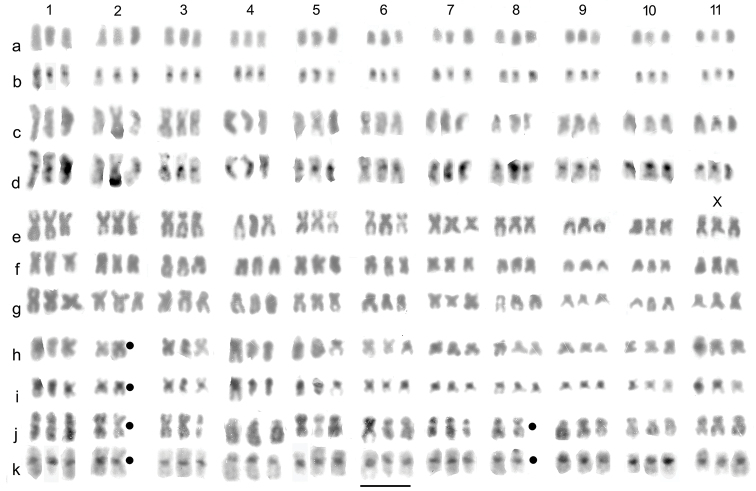
Karyotypes of *Helophorus* spp., females, preparations from mid gut. **a–d***H. orientalis***a, b** mishan **a** Giemsa-stained **b** the same nucleus C-banded **c, d** Qitaihe **c** Giemsa-stained **d** the same nucleus C-banded **e–k***H. brevipalpis***e, f** Algadefe, Giemsa-stained **e** from Angus (1992)**f** from a different female **g** from Ponte Scipione, Giemsa-stained **h–k** from Sologno **h** Giemsa-stained **i** the same nucleus C-banded **j, k** a preparation from a different female **j** Giemsa-stained **k** the same nucleus C-banded. All preparations from this locality lack one replicate of autosome 2, but the loss of an autosome 8 from **j** and **k** is experimental artefact. Scale bar: 5 µm.

The chromosome number of *H. orientalis* was found to be 3n = 33. Mitotic chromosomes, arranged as karyotypes, are shown in Fig. 1a–d. As no data on males are available, the X chromosome cannot be identified. All the chromosomes have distinct centromeric C-bands. There is a gradual decrease in length from chromosome 1 down to chromosome 7, which is about two thirds the length of chromosome 1. Chromosomes 8–11 are slightly smaller, about half the length of chromosome 1. Chromosomes 1–6 are metacentric, 9 is submetacentric and 7, 8 10 and 11 are subacrocentric. A consistent feature of the karyotype is that one replicate of chromosome 1 is consistently larger than the other two. C-banding suggests that the difference between the longer and shorter replicates may be associated with a weakly C-banding region towards the distal end of the long arm. In the more extended preparations (Fig. 1d) there is a second C-band at the distal end of the long arm (obscured by chromosome overlap in one replicate), but in the more contracted preparations (Fig. 1b) the terminal band appears smaller in the long replicate and does not show at all in the other two replicates.

### *H. brevipalpis* Bedel, 1881

The chromosome number of *H. brevipalpis* was found to be 3n = 33. Angus (1992) gave a detailed account of the chromosomes of both sexually reproducing diploid and parthenogenetic triploid *H. brevipalpis*, the triploid material coming from Algadefe (Spain, Provincia de León). Fig. 1e shows the specimen figured by Angus (1992) and Fig. 1f shows a karyogram from a different female. Both appear to show one replicate of autosome 1 shorter than the other two.

It is now possible to add data on Italian material. A specimen from the Provincia di Parma, analysed in 2008 (Angus and Foster 2009), has a karyotype closely resembling that of Spanish material, though the short replicate of autosome 1 is less obvious. Specimens from the Provincia di Reggio Emilia, analysed in the present study appear rather different. Data were obtained from five triploid females in the present study, and the highest chromosome number found was 32 in all cases. The resulting karyograms (Fig. 1h–k) show only two replicates of autosome 2. They also show autosome 3 to be more metacentric than in the Spanish and Parma material (Fig. 1e–g). The karyograms shown in Fig. 1j, k also lack one replicate of autosome 8. This is a preparation artefact as other preparations from this female have 32 chromosomes. This preparation is illustrated because it shows the form and C-banding of the chromosomes more clearly than the others.

## Discussion

**Figure 2.**
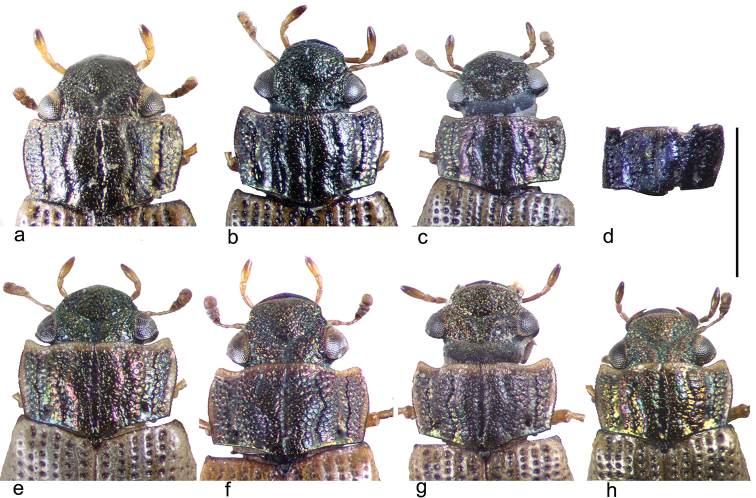
Heads and pronota of *Helophorus* species **a–d***H. orientalis***a** triploid female from Qitaihe **b** male from Logan **c** female from Waterloo, Ontario **d** fossil pronotum from Brandon Terrace, Warwickshire **e–h***H. brevipalpis*, parthenogenetic females **e** from Logan Canyon, Utah **f** triploid from Algadefe **g** triploid from Ponte Scipione **h** triploid from near Sologno. Scale bar: 1 mm.

**Figure 3.**
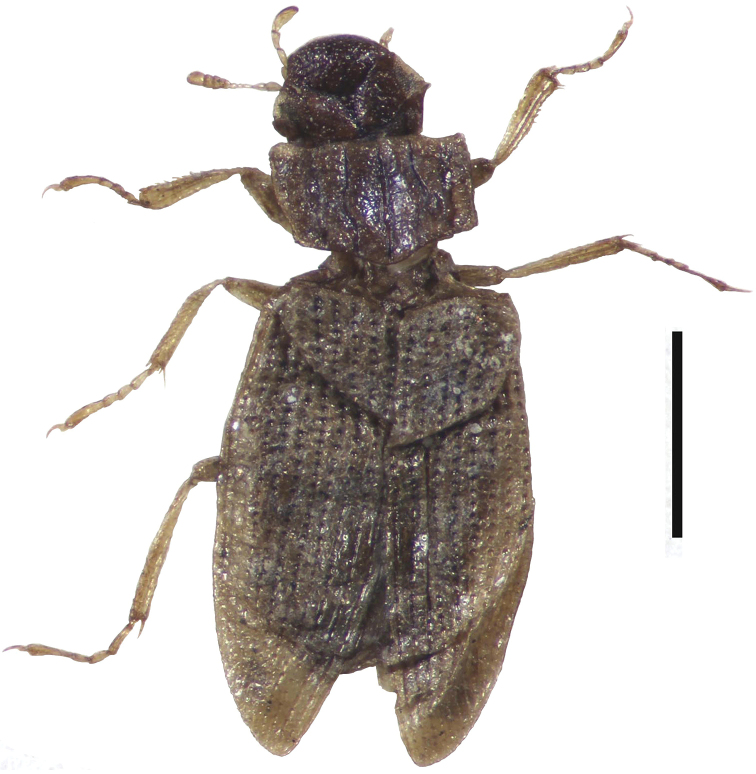
Fossil *H. orientalis* from Starunia. Scale bar: 1 mm.

As noted in the Introduction, *Helophorus orientalis* was the first *Helophorus* species shown to be parthenogenetic, following laboratory rearing by Angus of females sent to him in 1967 by Prof. C. H. Fernando from Waterloo, Ontario, Canada (Angus 1970). No males were present in this material. Smetana (1985) records male *H. orientalis* from the central Rocky Mountains of America, and there are males among material sent from Logan, Utah to the Natural History Museum in London. Smetana notes that in some populations from Wyoming males account for about 30% of the specimens. Apart from this American material, there are males in the collection of the late G. Lafer of Vladivostok, Russia. A sample from Novitskoye, 12 km south of Partizansk (about 80 km east of Vladivostok) comprised three males and seven females. A further sample from this area, from the village of Prudovoye in the Partizansk region, comprised 10 females, but no males. Lafer’s collection contains 38 females from other parts of the Russian Far East (Primorye), but no males. *H. orientalis* is abundant in East Siberia, but only as females. The picture emerging is of the species parthenogenetic over most of its wide distribution range, with bisexual populations in limited, separate areas. *H. orientalis* has a characteristic pronotum, with the internal intervals shining and with very reduced granulation. Fig. 2a shows this feature in a Chinese female, while Fig. 2b shows a Logan male and Fig. 2c shows a female from Waterloo. This pronotum is matched by an English Pleistocene fossil from Brandon, Warwickshire, with radiocarbon dates suggesting an age of about 30,000 years B.P. (Fig. 2d) (Coope 1968, Shotton 1968). *H. orientalis* is now known as a fossil from a number of English sites dating from the Last Glaciation, as well as from the classic Woolly Rhinoceros site at Starunia in the Western Ukraine, where more or less intact beetles may be found (Angus 1973) The Starunia rhinoceros has now been radiocarbon dated at about 33500–40000 years B.P. (Kuc et al. 2012). A fossil female from Starunia is shown in Fig. 3.

As already noted, the longest triplet of chromosomes includes one replicate which is distinctly longer than the other two, possibly associated with differing amounts of weakly C-banding material at the distal end of the long arm. This could suggest that these triploids have a hybrid origin. We know of no other species closely resembling *H. orientalis*, but with such a vast range and long fossil record indicating changes in its distribution, it is possible that different bisexual populations could be, or have been, sufficiently different genetically to cause some chromosomal mismatching if they hybridised. Experimental hybrids between *Helophorus lapponicus* Thomson, 1853 and *H. paraminutus* Angus, 1986 may be relevant here. Angus (1986), working at Karasuk, West Siberia, found that the karyotypes of these two species appeared indistinguishable, so he obtained experimental hybrids between them, with a view to having the chromosomes of the two species in exact synchrony in their condensation through mitotic prophase. The result, however, was some irregularities in their condensation, which Angus speculated might be the result of difficulty in uptake of non-histone protein by the condensing chromosomes. At the same time, crosses between Spanish and Swedish *H. lapponicus* resulted in no such irregularities.

The Spanish triploid *H. brevipalpis* also show chromosomal mismatching in the longest triplet, in this case involving one replicate being noticeably shorter than the other two, also shown by the Italian specimen from Parma province, Ponte Scipione. As with *H. orientalis*, *H. brevipalpis* is a distinctive species, but in this case also variable. Angus (1988) undertook detailed analysis of local populations of *H. brevipalpis*. This led to the recognition of a distinct subspecies from eastern Turkey, Syria and Iran. Discriminant functions analysis showed that this subspecies was more distinct from other populations than their variation among themselves, but also showed that these populations grouped into sections with slightly larger or smaller aedeagi. Spanish material comes in the slightly larger aedeagus group and Italian in the slightly smaller group. In terms of frequency of males, most Spanish material is clearly bisexual, and the males and diploid females found by Angus probably reflect the interface between the ranges of bisexual and parthenogenetic populations. It is also worth noting that, surprisingly, the province of Leon is at the edge of the range of *H. brevipalpis*, which is unknown in Spanish Galicia. What seems important here is that the regional variation found in *H. brevipalpis* allows the possibility that the triploids may have resulted from crossing between genetically differing stocks.

The Italian triploids from Sologno differ from the others encountered in having the autosomes of triplet 3 more evenly metacentric than in the other triploids, and in having only two replicates of autosome 2. The difference in triplet 3 must reflect origin from a different bisexual stock, while the loss of one replicate of autosome 2 presumably results from an “accident”. In the absence of knowledge of the oogenesis of this species it is not possible to say whether the parthenogenesis has always been apomictic (without any meiosis) or whether there might be automictic parthenogenesis (with meiosis followed by fusion of haploid oocytes) in some diploid females. Apomictic parthenogenesis would result in offspring that are clones of the parent, while automictic parthenogenesis would allow limited variation, and, perhaps, account for the loss of a chromosome.

Parthenogenesis in *Anacaena lutescens* has taken a different course. Shaarawi and Angus (1991) analysed the chromosomes and found that, unlike the situation in *Helophorus*, most parthenogenetic material was diploid, and carried a heterozygous deletion of a small apical section of autosome 8, distal to a secondary constriction. Two triploid populations were found, one from Cumbria (England) and one from the Netherlands. However, these populations differed in the arrangement of autosome 8, indicating that their triploidy evolved independently, after the onset of parthenogenesis.

In all these cases, parthenogenesis was initially suspected on the basis of under-representation of males in sampled material. At the moment, we know of one further *Helophorus* species in which this might also be the case. *Helophorus aquila* Angus et al., 2014 was described from the northern part of Qinghai (China). Only 3 males were taken among more than 80 specimens. This area, near the great lake of Qinghai Hu, is readily accessible so this should be verified in further studies.
